# Dually Fluorescent Core-Shell Microgels for Ratiometric Imaging in Live Antigen-Presenting Cells

**DOI:** 10.1371/journal.pone.0088185

**Published:** 2014-02-04

**Authors:** Xianfeng Zhou, Fengyu Su, Yanqing Tian, Deirdre R. Meldrum

**Affiliations:** Center for Biosignatures Discovery Automation, Biodesign Institute, Arizona State University, Tempe, Arrizona, United States of America; RMIT University, Australia

## Abstract

Core-shell microgels containing sensors/dyes in a matrix were fabricated by two-stage free radical precipitation polymerization method for ratiometric sensing/imaging. The microgels composing of poly(*N*-isopropylacrylamide) (PNIPAm) shell exhibits a low critical solution temperature (LCST), underwent an entropically driven transition from a swollen state to a deswollen state, which exhibit a hydrodynamic radius of ∼450 nm at 25°C (in vitro) and ∼190 nm at 37°C (*in vivo*). The microgel’s ability of escaping from lysosome into cytosol makes the microgel be a potential candidate for cytosolic delivery of sensors/probes. Non-invasive imaging/sensing in Antigen-presenting cells (APCs) was feasible by monitoring the changes of fluorescence intensity ratios. Thus, these biocompatible microgels-based imaging/sensing agents may be expected to expand current molecular imaging/sensing techniques into methods applicable to studies in vivo, which could further drive APC-based treatments.

## Introduction

Antigen-presenting cells (APCs) internalize antigens, present antigen-derived peptides to T cells containing major histocompatibility complexes (MHCs) on their surfaces, and play a pivotal role in both initiation and regulation of immune responses [1]. Intracellular variables such as pH and oxygen levels are important factors in regulation of the antigen-presenting processes [2]. The interferon-gamma (IFN-γ) and pro-inflammatory cytokine produced by APCs is dictated by intracellular local oxygen tension [3]; therefore, there is much interest in the development of methods suitable for detection of essential analytes (such as dissolved oxygen and pH) in the clinical sitting of APCs. The development of cellular imaging and sensing techniques is imperative to ultimately advancing APCs-based therapy. Fluorescent sensor-based imaging of cells is an alternative, noninvasive imaging modality with the capability of cellular events tracking [4]–[6]. The approach has intrinsic value because asynchronous and single-cell level behaviors of APCs are not indicated by population measurements [7].

Polymer-based fluorescent nanosensors [8], [9], or probes encapsulated by biologically localized embedding sensors (PEBBLEs), were first created to be used for intracellular measurements by creating a biocompatible shell around the probes [10], [11]. The real power of particle-based nanosensors was realized when PEBBLEs were embedded with a sensing probe and a reference dye [12]. This feature makes the sensor ratiometric. By plotting the ratio of sensing probe over reference dye emission *vs.* analyte concentration, the fluorescence signal can be handled in a quantitative manner. For these early sensors, however, the optical probes were physically incorporated into the polymer matrix. The possible leaching of these probes from the matrices might be a significant problem which may result in signal instability, inaccuracy of the measurement, decreased long-term applicability, and potential cytotoxicity for cells [13]. To address this problem, the probes/dyes have been covalently attached to the matrix to give sensing/imaging microgels [14], [15].

APCs can take up nanoparticles by ligand-mediated endocytosis. However, many other cell types also are capable of ingesting these small-sized particulate matters (typically <200 nm in diameter) by mechanisms such as pinocytosis and endocytosis [16]. The uptake of particles with diameters up to several micrometers is generally restricted to phagocytic APCs; therefore, tuning the size of particulate delivery systems can enable passive targeting of optical sensors to APCs. Several reports have highlighted the impact that particle sizes may have interactions with APCs [17]. For example, early studies on the functional application of particulate carriers indicated that carriers with diameters between 0.5 and 3 µm were effective for APCs *in vitro* [18]. However, *in vivo* studies using a similar polystyrene-based system suggest that particles less than 200 nm in diameter are effective at activating APCs [19].

**Figure 1:**
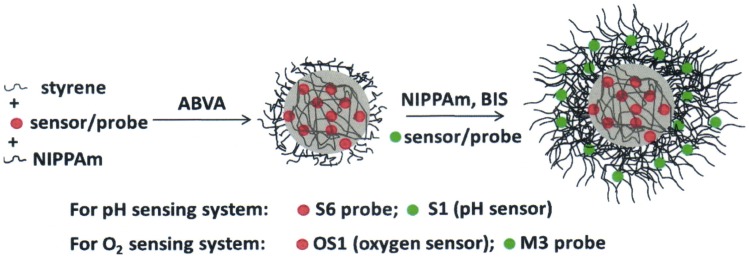
Schematic illustration of the preparation process of the P(St-*co*-NIPAm)-PNIPAm core-shell microgels.

Herein, we report on the fabrication of core-shell microgels with covalently incorporated sensors/dyes in the matrix for ratiometric pH and oxygen sensing (Figure 1). The microgels are mainly composed of a polystyrene (PSt) core and a poly(*N*-isopropylacrylamide) (PNIPAm) shell. Such a structure makes it possible for the microgels to be stable in aqueous solutions and act as efficient solubilizers of hydrophobic sensors/dyes to enable their applications in biological environments. This versatile matrix allows for chemical immobilization of the probes either into the core or the shell for ratiometric imaging/sensing. Furthermore, we found that these PNIPAm microgels exhibited a low critical solution temperature (LCST) or volume phase transition (VPT) temperature of ∼32°C in buffer (Figure S1). At this temperature, the microgels undergo an entropically driven transition from a swollen state to a deswollen state [20], leading to the facile modulation of the hydrodynamic radius ∼500 nm at 25°C (*T*<LCST) and ∼190 nm at 37°C (*T*>LCST). Based on this, the diameter of 190 nm may be expected for use in a living model in the future at 37°C. We also investigated the bio-imaging application of PSt/PNIPAm (core/shell) microgels in APCs. Of critical importance, the selective uptake was reproduced using macrophages (J774A.1 cells) versus cervical cancer cells (Hela cells). The result of MTT viability assay showed that the novel microgels are almost noncytotoxic with a concentration up to 10 mg/mL. All these results well support the applications of microgels in APCs for multifunctional purposes such as sensing and imaging.

## Experimental Section

### Materials

NIPAm monomer was purified by recrystallization from n-hexane. Styrene (St) was distilled under reduced pressure prior to use. Other chemicals and solvents were of analytical grade and were used without further purification. Milli-Q water (18 MΩ) was used for all the titrations. Calibration gases (nitrogen and oxygen) were purchased from AIR Liquide America, LP (Houston, TX). Exact gas percentage was precisely controlled with a custom-built, in-line, digital gas flow controller. The pH values were determined with a digital pH meter (Thermo Electron Corporation, Beverly, MA) calibrated at room temperature (23±2°C) with standard buffers of pH 10.01, 7.00, and 4.01. All sensing measurements were carried out at atmospheric pressure, 760 mmHg or 101.3 kPa. Hoechst 33342, and LysoTracker Red® were purchased from Invitrogen (Carlsbad, CA). Eagle’s minimal essential medium (EMEM) was used for HeLa and J774A.1 cells culture. The pH sensing monomer (S1), oxygen sensing monomer (OS1), and the reference probes M3 and S6 were synthesized according to our published literature (Figure S2) [21]–[23].

### Instruments

Polymer microgel sizes were measured using a 173° back-scattering Zetasizer Nano-ZS (Malvern Instruments, Worcestershire, United Kingdom). A JEOL 1010 transmission electron microscope (TEM) operated at 100 KV was employed to obtain TEM images. The microscope samples were prepared by placing a drop of the polymer dispersion on a carbon-coated Cu grid, followed by solvent evaporation at room temperature. All spectroscopic measurements were carried out with a 10-mm-path-length quartz cell. Fluorescence spectra were measured on a Shimadzu RF-5301 spectrofluorophotometer (Shimadzu Scientific Instruments, Columbia, MD).

### Preparation of the Core-shell Microgels

The core-shell microgels were prepared according to the literature procedure with some modifications [24]. The polymerization was carried out in a 250 mL three-necked flask equipped with a nitrogen inlet, a stirrer, and a condenser. First, P(St-co-NIPAm) cores were prepared by emulsion polymerization. Briefly, NIPAm, St, surfactant Tween 20 and sensor/probe were dissolved in 95 mL of water. Then the mixture was stirred and nitrogen was bubbled into the mixture for 30 min. After the temperature increased to 70°C, 4,4′-azobis(4-cyano-valeric acid) (ABVA) dissolved in 5 mL of water was injected to initiate the polymerization. The PNIPAm shell layers were fabricated on the P(St-co-NIPAm) cores by a seeded emulsion polymerization. In brief, 25 mL of the core solution was taken in a three-necked round bottom flask, to which Tween 20 and 65 ml of water were added. The solution was heated under nitrogen to 70°C. NIPAm, *N,N’*-methylene bisacrylamide (BIS) and *N*-(3-aminopropyl) methacrylamide hydrochloride (APMA) were dissolved in 10 mL of water and degassed at room temperature for 1 h and then added to the heated core solution. Finally, ABVA dissolved in 1 mL of water was added to the solution to initiate the reaction. The reaction was allowed to proceed for 6 h at 70°C and then was cooled. The microgels were then dialyzed for one week against daily changes of PBS buffer (pH 7.4).

The S1 NIPAm microgels were prepared according to the literature procedure with some modifications [25]. In brief, NIPAm, BIS, surfactant Tween 20 and S1 were dissolved in 95 mL of water. Then the mixture was stirred and nitrogen was bubbled into the mixture for 30 min. After the temperature increased to 70°C, 4,4′-azobis(4-cyano-valeric acid) (ABVA) dissolved in 5 mL of water was injected to initiate the polymerization. The reaction was allowed to proceed for 4 hours at 70°C and then was cooled. The solution was then dialyzed for one week against daily changes of PBS buffer (pH 7.4).

### Culture of Cells for Intracellular Imaging

Murine macrophages J774A.1 (ATCC® TIB-67™, Manassas, VA) were cultured in EMEM supplemented with 10% fetal bovine serum, 100 u/mL penicillin, 2 mM L-glutamine (Sigma), and incubated at 37°C in a 5% CO_2_ atmosphere. For the phagocytosis experiment, macrophages were incubated with 100 µl of microgels (1 mg/mL) overnight in EMEM. The macrophages were then washed three times with a PBS buffer to remove excess microgels. To confirm the subcellular distribution of microgels, LysoTracker Red® was added to co-stain lysosomes. Cells were incubated for an additional 30 min for observation of colocalization of the microsgels and the LyoTracker Red®. The medium was removed and the cells were washed once with cold phosphate buffered saline (PBS). Hoechst 33342 dissolved in fresh medium was then added into the medium to stain cell nuclei for 30 min. Concentrations of LysoTracker Red® and Hoechst 33342 were 100 nM and 1 µM, respectively. Under Nikon Eclipse TE2000E confocal fluorescence microscope (Melville, NY), Hoechst 33342 was excited at 402 nm and its blue emission was collected using a 450/35 nm filter set; microgels were excited at 440 nm and their green emissions were collected using a 515/30 nm filter set; LysoTracker Red® was excited at 561 nm and its red emission was collected using a 605/75 nm filter set. As the control, HeLa cells (ATCC® CCL-2™) were cultured in EMEM supplemented with 10% fetal bovine serum, 5% penicillin, 2 mM L-glutamine (Sigma), and incubated at 37°C in a 5% CO_2_ atmosphere. Then HeLa cells were incubated with microgels for 24 h.

## Results and Discussion

**Figure 2:**
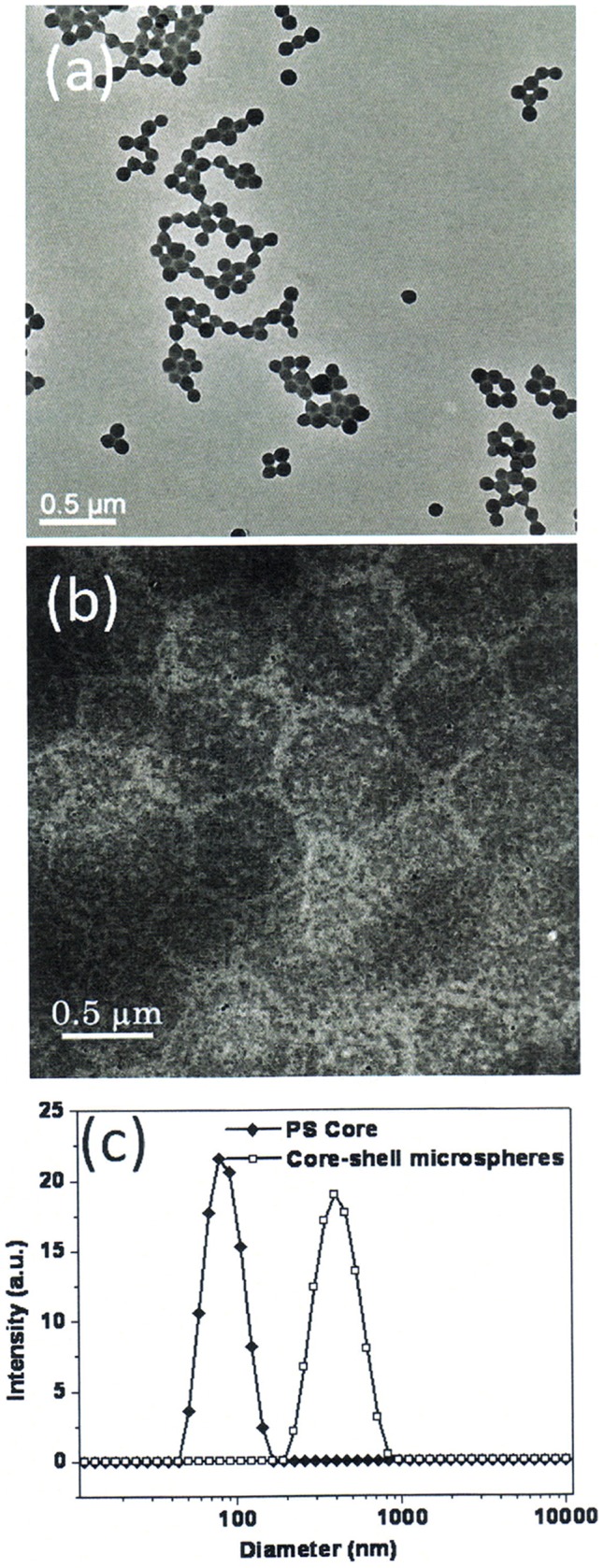
Size and distribution of the core (OS1 in PSt) and the core-shell microgels (MS1) prepared by microemulsion polymerization determined by TEM (a, b) and DLS (c).

Firstly, we optimized the size of the microgel by altering the compositions of either the core or the shell (supporting information, Table S1, Table S2, and Table S3). Figure 2 shows a size distribution of the representative PSt core with a platinum porphyrin based oxygen sensor of OS1 (Figure S2) and core-shell microgels of MS1 with a reference fluorophore of M3 (Figure S2) in the PNIPAm shell determined by dynamic light scattering (DLS) and transmission electron microscopy (TEM). The hydrodynamic size of the PSt core varies from 50 to 140 nm with an average diameter of 95 nm. The hydrodynamic size of the core-shell microgels varies from 200 to 800 nm with an average diameter of 500 nm.

### Microgels for Sensing Oxygen

**Figure 3:**
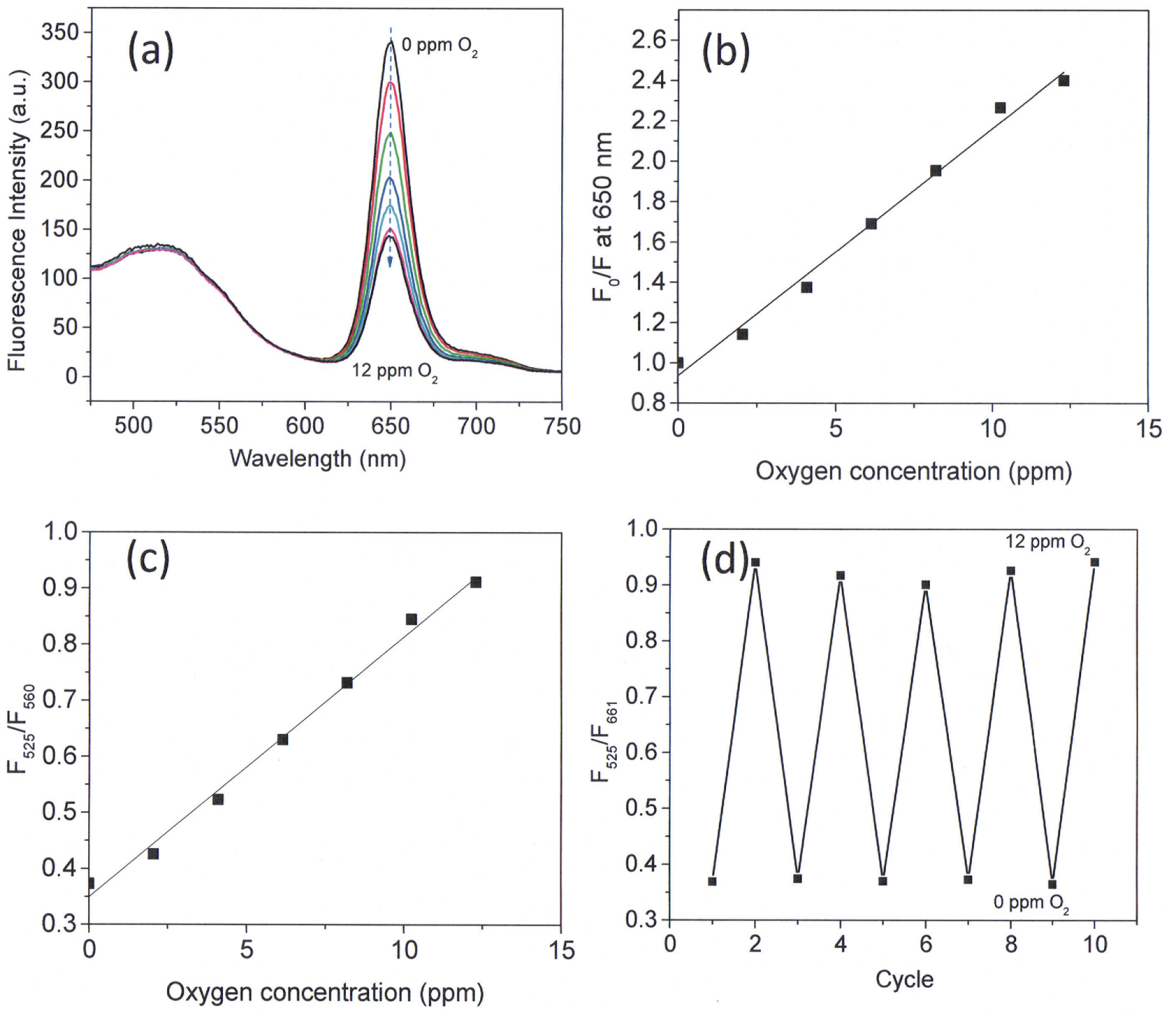
Response of the core-shell microgels (MS1) to dissolved oxygen in PBS buffer. (a) Typical response to dissolved oxygen in PBS pH 7.4 buffer. (b–c) Fits of the Stern-Volmer plots, whichwere performed using eq 1 with/without ratiometric calibration. (d) Change in fluorescence intensity ratio (F_525_/F_650_) of MS1 in PBS buffer, where the oxygen concentration was changed repeatedly between 0 and 12 ppm.

OS1 was chosen as an oxygen-sensitive probe because of its excellent photostability and good brightness. The oxygen sensor is a monomer, which emits in the red spectral window. The OS1 was polymerized with another monomer (herein styrene) to form OS1-containing PSt cores. An oxygen-insensitive green emitter (M3) was chosen as a reference probe and was polymerized into the PNIPAm shell. Figure 3a shows the oxygen response of the OS1/M3 (core/shell) microgel (MS1) measured in buffer at ambient temperature. A marked dependence of fluorescence intensity on dissolved oxygen concentrations [O_2_] was observed, showing that the emission of the oxygen probe was physically quenched by oxygen. The oxygen quenching process is ideally described by the linear Stern-Volmer equation:
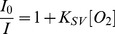
(1)where *K*_SV_ is the Stern-Volmer quenching constant and [O*_2_*] is the dissolved oxygen concentration. At 23°C, [O_2_] in water is 8.57 ppm at atmospheric pressure corresponding to an oxygen partial pressure of 21 kPa. *I_0_* and *I* are the steady-state fluorescence signals measured in the presence of nitrogen and various oxygen concentrations generated by controlled gas bubbling, respectively. The fluorescence intensity ratio in the absence and presence of oxygen at 12 ppm (30% of oxygen in the mixture of oxygen and nitrogen), *I*_0_/*I*_12_, was approximately 2.4 at room temperature with a *K_SV_* of 0.126 ppm^−1^ (R^2^>0.988). This data is in agreement with PSt thin hydrogel film oxygen sensor designed in our group [26]. The calibration curve for the ratiometric microgels shows the response to dissolved oxygen as displayed in Figure 3c. Fluorescence emission intensity maximums of amino-naphthalimide of M3 (525 nm) and platinum porphyrin of OS1 (650 nm) were used to determine the ratios (λ_exc_ = 402 nm). The linearity (R^2^>0.992) of the Stern-Volmer plot gave a *K_SV_* ^’^ of 0.127, which implies that a single probe class is accessible to molecular oxygen [27]. Many oxygen sensing films, hydrogels, and silica particles consisting of the oxygen sensors trapped in the matrix do not have linear Stern-Volmer constants. The non-linearity of the Stern-Volmer plot is a result of some probe molecules lacking oxygen because of the inability of oxygen to penetrate into the matrix [28]. In other words, the MS1 microgel allows the oxygen to interact uniformly with a greater proportion of probes, thus resulting in a linear range in the Stern-Volmer plot. Figure 3d shows the change of the fluorescence intensity ratio (F_525_/F_650_), where the dissolved oxygen concentration is changed repeatedly between 0 and 12 ppm. The data showed that the reversibility of the sensor for at least 5 cycles.

### Microgels for pH Sensing

**Figure 4:**
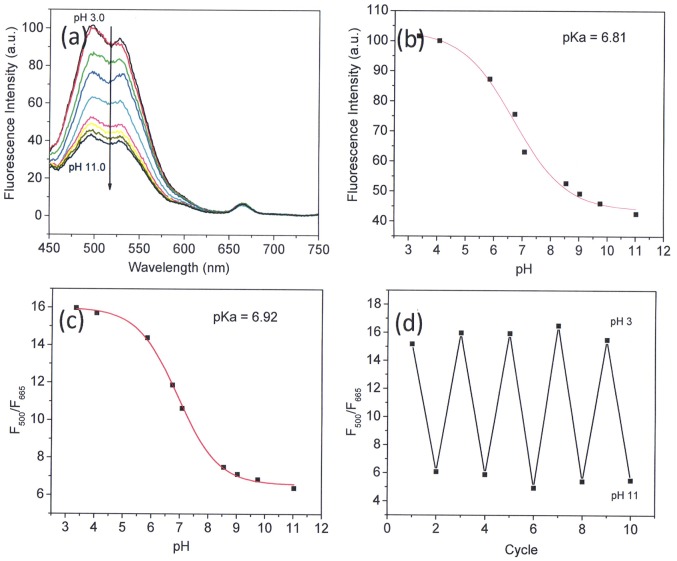
Response of the core-shell microgels (MS2) to pH in PBS buffer. (a) Typical fluorescence intensity change at different pH values in PBS buffer. (b–c) Boltzmann fittings, which were performed using eq 2 with/without ratiometric calibration. (d) Change in fluorescence intensity ratio (F_500_/F_665_) of MS2 in PBS buffer, where the pH was changed repeatedly between 3 and 11.

An amino-naphthalimide-based monomeric compound S1 [4] (Figure S2) was chosen as a typical pH sensor. It was polymerized into the PNIPAm shell. A pH-insensitive red emitter (S6 [21], Figure S2) was chosen as a reference dye and was polymerized into the PSt core. Figure 4a shows the emission spectra of the S6/S1 (core/shell) microgels (MS2) in PBS buffers at different pH. Fluorescence intensity increased with a decrease in pH value. The fluorescence intensity changes are described well by a sigmoidal function (Boltzmann fitting) as shown in equation 2.
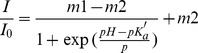
(2)where, *I* and *I*_0_ are the fluorescence intensities measured at varying pH values and at the highest pH value used during the calibration, respectively. Empirical parameters, *m*1, *m*2, p*K*_a_^’^, and *p* describe the initial value (*m*1), the final value (*m*2), the point of inflection (p*K*_a_^’^), and the width (*p*) of the sigmoidal curve. The fluorescence intensity changes at 500 nm and their curve fittings are shown in Figure 4b. The apparent p*K*_a_ value was 6.81 for the microgels (MS2) in PBS buffers. The fitting was reliable with a correlation coefficient (R^2^) of 0.992. Figure 4c shows the ratios of fluorescence intensities at 500 and 665 nm (λ_exc_ = 402 nm) at different pH. A minor change is observed with the apparent p*K*_a_ value 6.92 for the microgels (MS2) at ratiometric sensing mode as compared to the *pK*_a_ value of 6.91 using the pH sensor only, suggesting that the MS2 microgels be suitable for pH measurements in physiological conditions. It should be noted here that the sensitivity (*F_max_*/*F_min_* = 2.4) of MS2 decreased compared with that of S1 only in PNIPAm microgels (*F_max_*/*F_min_* = 6.0, Figure S3). This may be due to the fluorescence energy resonance transfer (FRET) from S1 to S6 or the aggregation quenching of S1 [29]. Figure 4d shows the change of the fluorescence intensity ratios (F_500_/F_665_), where the pH is changed repeatedly between 3 and 11. The data clearly shows that the fluorescence ratio (F_500_/F_665_) is reversibly changed at least 10 times.

### Cellular uptake

**Figure 5:**
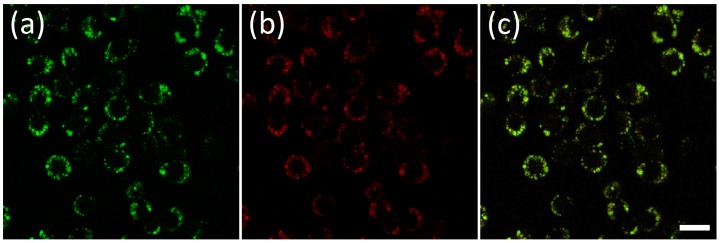
Confocal microscopy images of J774A.1 cells treated with core-shell microgel MS1. (a) Naphthalimide green fluorescence of core-shell microgels inside cells. (b) Porphyrin red fluorescence of core-shell microgels inside cells. (c) Overlay images of (a) and (b). Green fluorescence was excited at 440 nm and emissions were collected using a 515/30 nm filter set; Red fluorescence was excited at 440 nm and its red emission was collected using a 605/75 nm filter set. Scale bars represent 20 µm.

Murine macrophages J774A.1 (American Type Culture Collection, ATCC, Manassas, VA) were chosen to investigate the cellular uptake of the core-shell microgels. Macrophages are the professional APCs which are most prominent in inflammatory sites and are specialized for clearing necrotic and apoptotic materials. Furthermore, macrophages can also play either pro- or anti-inflammatory roles, depending on the means by which they are activated [30]. The core-shell microgels (MS1) were incubated with J774A.1 cells. Confocal fluorescent microscopic images showed that the green fluorescence from the M3 segment is colocalized completely with the red fluorescence from OS1 moieties (Pearson’s sample correlation factors, R_r_ >99.1%) [31]. This indicated that the core-shell microgels embedded with two fluorophores were taken up by macrophages and the core-shell microgels were localized in cells (Figure 5). In order to avoid confusion, when we performed the colocalization study, we only use the fluorescence in the green channel to represent the microgels.

**Figure 6:**
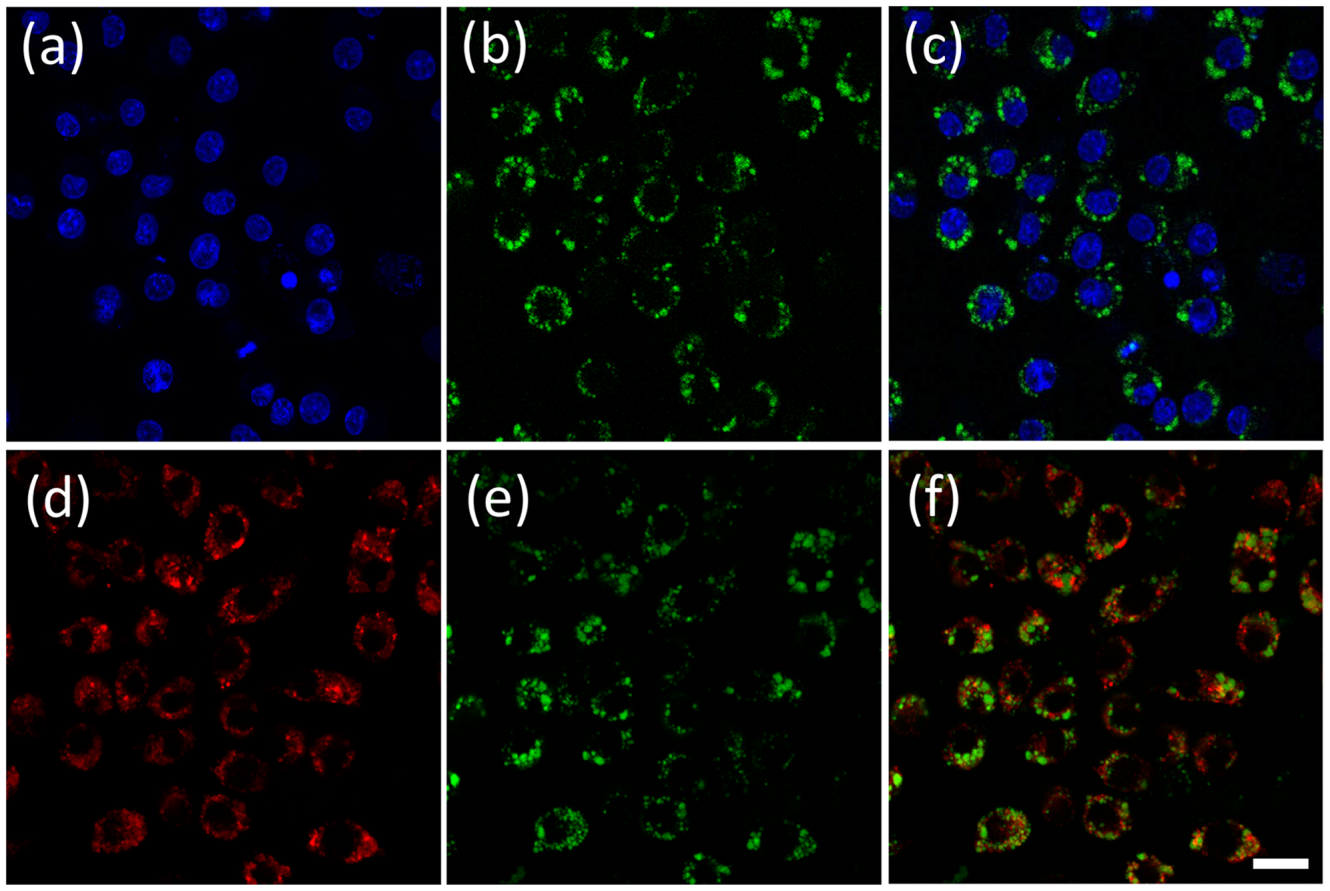
Confocal microscopy images of core-shell microgels MS1 in J774A.1 cells co-stained with nucleic staining Hoechst 33342 (a–c) and LysoTracker Red® (d–f). (a) Hoechst 33342 blue fluorescence inside cells. (b) and (e) Green fluorescence of core-shell microgels inside cells. (d) LysoTracker Red® red fluorescence inside cells. (c) Overlay images of (a) and (b). (f) Overlay images of (d) and (e). Hoechst 33342 was excited at 402 nm and its blue emission was collected using a 450/35 nm filter set; green fluorescence was excited at 440 nm and emissions were collected using a 515/30 nm filter set; LysoTracker Red® fluorescence was excited at 561 nm and its red emission was collected using a 605/75 nm filter set. Scale bars represent 20 µm.

To determine the subcellular distributions of microgels, a secondary dye-staining method was used to track the nucleus and the acidic compartments in the cells. First of all, the nuclei specific staining probe Hoechst 33342 was used to co-stain the cells with core-shell microgels. Small spherical green emissions distributed mainly in the cytoplasm region were observed under confocal microscopy, which was confirmed because of only minimal colocalization of the green emissions (from microgels) with blue emissions (from Hoechst 33342) (Figure 6a–c). Cells were also co-stained using a commercially available lysosome specific staining probe LysoTracker Red® and the core-shell microgel. It is evident that significant amount of the microgels lie outside of the lysosomes, since the green fluorescence is largely anticorrelated with the red fluorescence channel (Figure 6d–f). A magnified high resolution Figure 6f was given in the supplementary information as Figure S4. This result can be further confirmed from the colocalization efficiency calculation. The Pearson’s sample correlation factor is 56% and overlap coefficient is 62%. This behavior of the microgels, that they were phagocytosed but were not retained in the lysosomes, greatly increases the potential applicability of these microgels for cytosolic sensing/imaging. Similar behavior has been observed previously for block copolymer micelles [32], [33]. For most particulate carriers, it is generally assumed that a triggering mechanism must occur in the lysosome to release the particulate matters in the cytosol [34]. Cationic lipids may possess some intrinsic bilayer-disrupting property, especially when forming non-lamellar phase (e.g. lipopolyamines from direct hexagonal phase [35]). Cationic polymers possess no fusogenic property and this is why polylysine and similar polymers require chloroquine, a lysosomotropic drug used to unmask the intravacuolar malaria parasite, to become an effective agent for cytosol delivery [36]. Interestingly, our microgels lack any purposefully designed mechanism and are located in the cytosol after being phagocyted. Hence, the structures of the microgels have potentials to be used as carriers for cytosolic delivery of sensors to cells.

**Figure 7:**
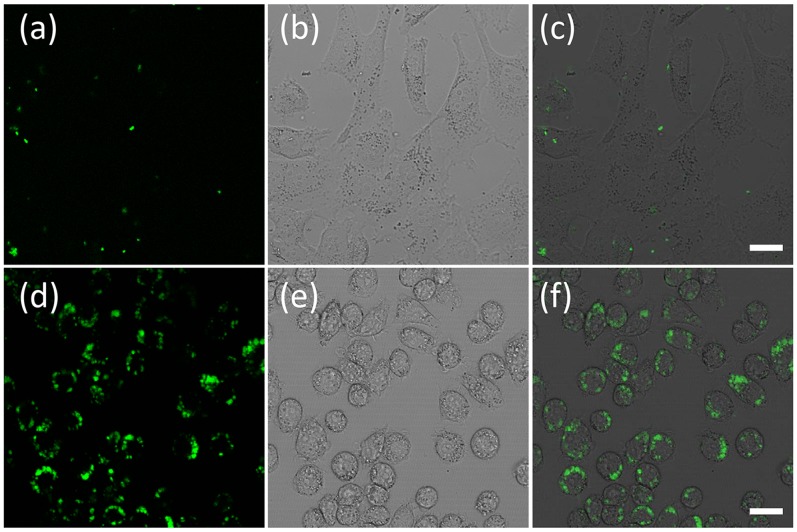
Confocal microscopy images of core-shell microgels MS1 in Hela cells (a–c, human cervical cancer) or J774A.1 cells (d–f, Mouse macrophage). (a) and (d) Green fluorescence of core-shell microgels inside cells. (b) and (e) Bright field images of cells. (c) and (d) Overlay images of (a)/(b) and (d)/(e). Green fluorescence was excited at 440 nm and emissions were collected using a 515/30 nm filter set. Scale bars represent 20 µm.

To investigate whether the particles could be taken up by other non-APCs, cervical cancer HeLa cells were used as counterparts. After 24 hours of cellular internalization using HeLa cells, only slight fluorescence was observed (Figure 7). These results suggest that cellular uptake of the microgels by APCs is through phagocytosis.

**Figure 8:**
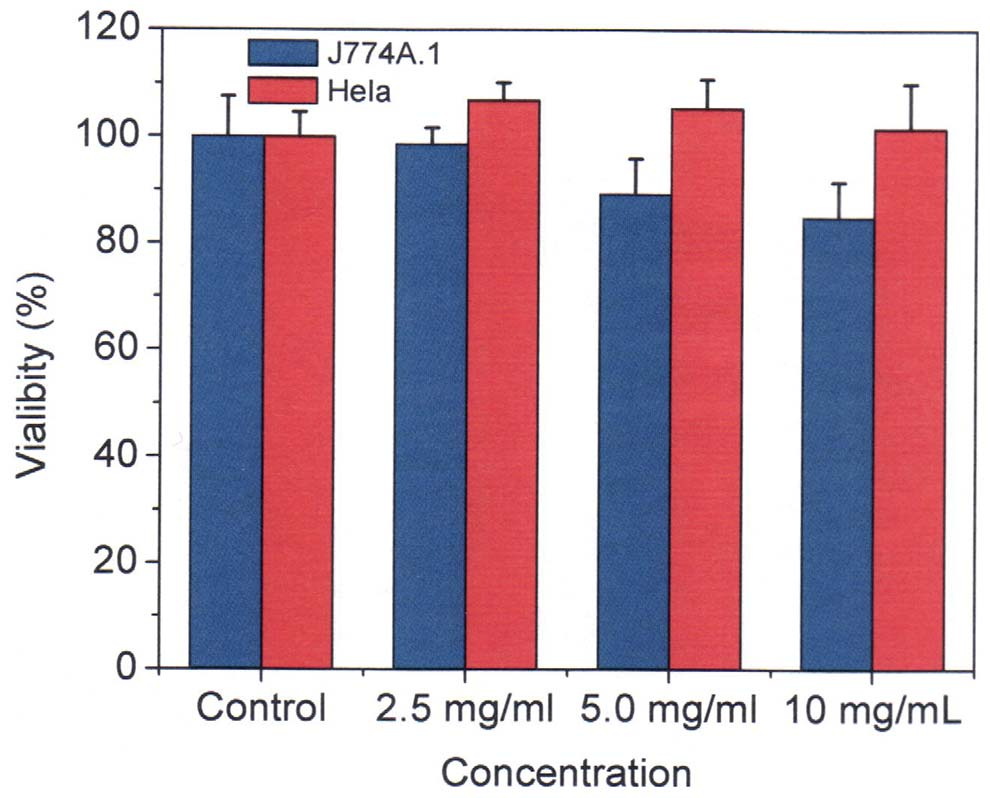
Cytotoxicity of the microgels MS1 to J774A.1 (blue) and Hela (red) cells after incubation at 37 °**C for 24 h.** The concentration of “control” in the x-axis means the control cells without adding any of the microgels**.**

Microgel-induced cytotoxicity was investigated by evaluating the cell viability. Cytotoxicity was determined as a function of concentration of the microgels by a standard MTT cell viability assay. More than 85% of the cells were viable after the cells were incubated for 24 h with the core-shell microgels concentrations of 2.5–10 mg/mL (Figure 8). These observations demonstrated the biocompatibility of the core-shell microgels.

## Conclusions

In summary, biocompatible core-shell microgels containing optical sensors/reference probes as a novel ratiometric imaging/sensing system for APCs was developed. The microgels can easily be prepared using the two-stage free radical precipitation polymerization method. The sensors/probes can be immobilized either into the core or into the shell. In our lab we have been developing new fluorescent sensors including pH, O_2_, Zn^2+^, DNA, and temperature sensors [4], [26], [37]–[44] for not only new materials but also applications for intracellular and extracellular sensing, especially at the single cell level [43], [44]. Our long term goal is to investigate cellular metabolism, disease/cancer detection and diagnosis using multi-sensor platforms. Herein, the core-shell microgels can have the potential for optical sensing and imaging of important analytes, such as O_2_, pH, and/or temperature. Furthermore, simultaneous sensing of two analytes (pH and oxygen) is also possible using the core-shell microgels platform (Figure S5). The ability of the microgels to escape from the lysosome into the cytosol makes them a potential candidate for cytosolic delivery of sensors/probes. With the help of the microgels, the noninvasive imaging/sensing in APCs was feasible. The biocompatible microgel-based imaging/sensing agents are expected to expand current molecular imaging techniques into methods applicable to studies *in vivo*, which will further drive APCs-based treatments.

## Supporting Information

Figure S1 **Temperature-dependent change in the hydrodynamic radius (R_h_) of the microgels measured by laser scattering analysis. The sigmoidal fitting was performed for LCST determination (32.2°C).**
(TIF)

Figure S2 **Structures of oxygen sensor (OS1) and pH sensor (S1). Oxygen-insensitive green dye (M3) and pH-insensitive red dye (S6) were used as the references for ratiometric sensing.** Red background represents the probe exhibiting red emission. Green background represents the probe exhibiting green emission.
(TIF)

Figure S3 **Response of the S1 in PNIPAm microgels to pH in PBS buffer.**
(TIF)

Figure S4 **Magnified** **Figure 6f** **.**
(TIF)

Figure S5 **Response to dissolved oxygen in buffer. Fits of the Stern-Volmer plot with/without ratiometric calibration.** (d–f) The emission profile of core-shell microgels changes as a function of pH. Intensity ratio variations with pH with/without ratiometric calibration.
(TIF)

Table S1 **Size of Poly(St-** ***co*** **-NIPAm) core (OS1) particles obtained by DLS at 25**°**C. In the systematic preparation of Poly(St-** ***co*** **-NIPAm) core particles, the dosages of St, NIPAm, and ABVA were kept constant as 1.0 g, 0.2 g and 0.01 mM, respectively.**
(DOC)

Table S2 **Hydrodynamic mean diameters of the core-shell microgels (MS1) prepared with different NIPAm dosages. For all samples, the w/w percentage of BIS is 3%.**
(DOC)

Table S3 **Hydrodynamic diameters of the core-shell microgels (MS1) prepared with different BIS dosages. For all samples, the dosage of NIPAm is 400 mg.**
(DOC)
