# Supplementary figures and images for: Dually Fluorescent Core-Shell Microgels for Ratiometric Imaging in Live Antigen-Presenting Cells
Source: PLoS One. 2014 Feb 4;9(2):e88185. doi: 10.1371/journal.pone.0088185 (PMC3913776; doi:10.1371/journal.pone.0088185)

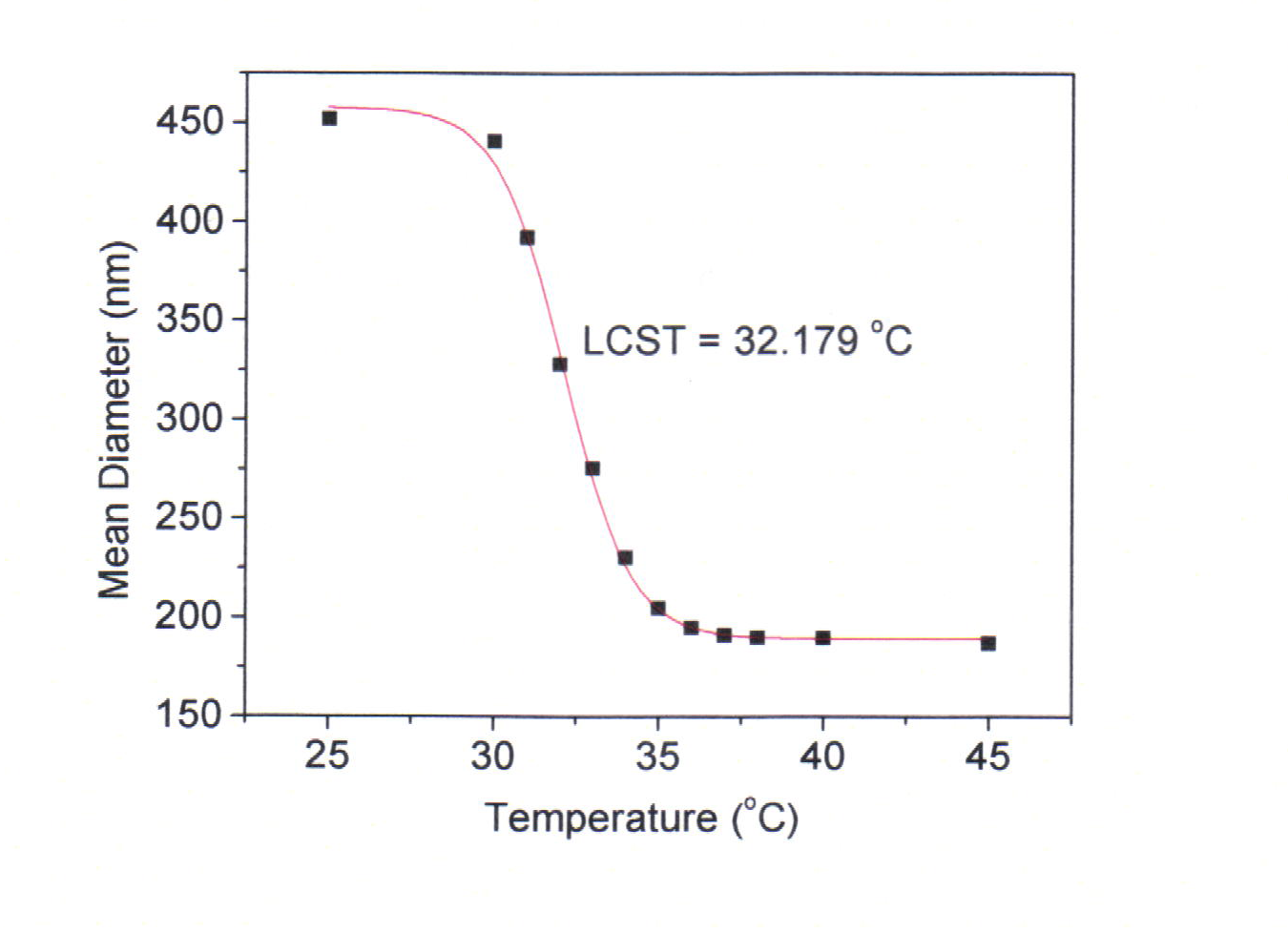

Supplement: Figure S1 — Temperature-dependent change in the hydrodynamic radius (Rh) of the microgels measured by laser scattering analysis. The sigmoidal fitting was performed for LCST determination (32.2°C). (TIF) [file pone.0088185.s001.tif]

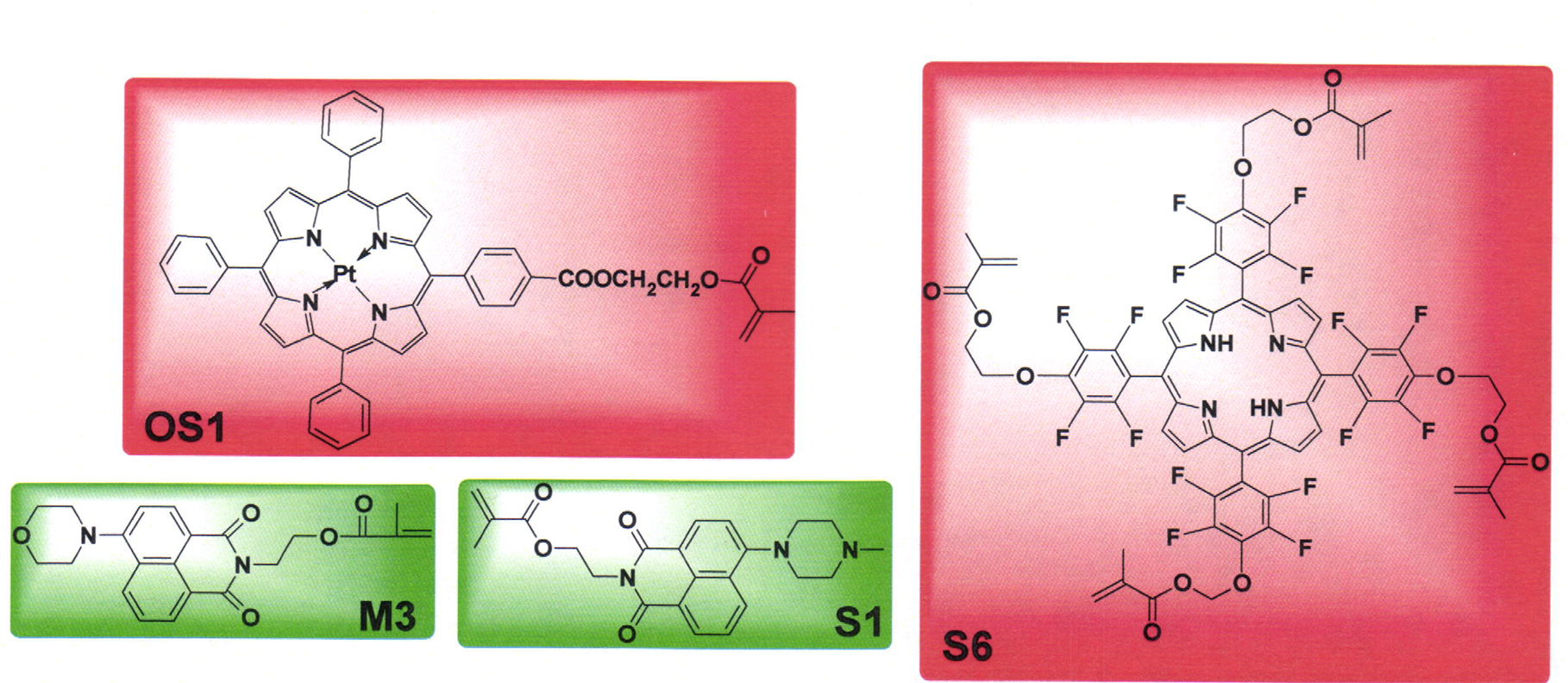

Supplement: Figure S2 — Structures of oxygen sensor (OS1) and pH sensor (S1). Oxygen-insensitive green dye (M3) and pH-insensitive red dye (S6) were used as the references for ratiometric sensing. Red background represents the probe exhibiting red emission. Green background represents the probe exhibiting green emission. (TIF) [file pone.0088185.s002.tif]

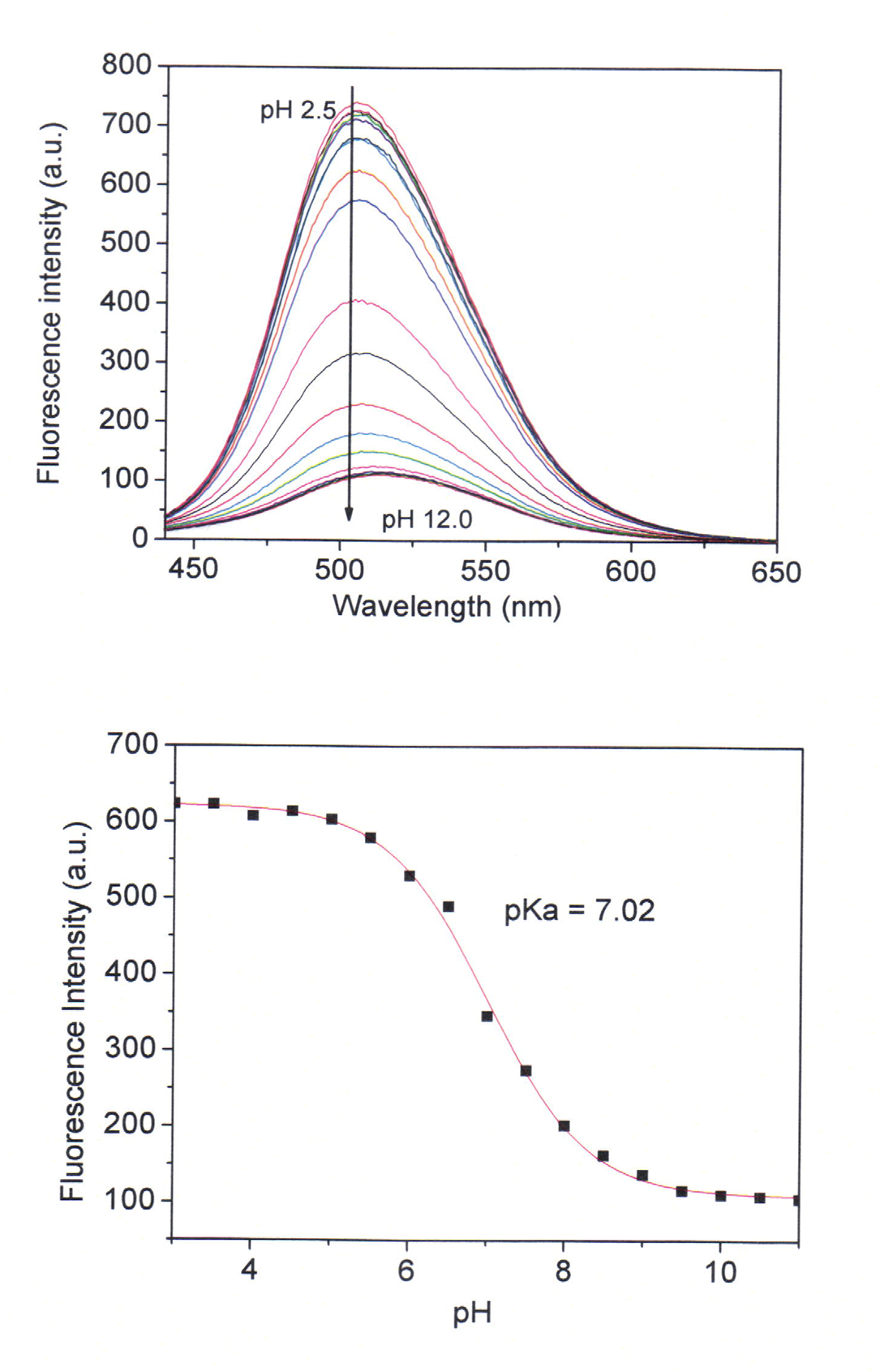

Supplement: Figure S3 — Response of the S1 in PNIPAm microgels to pH in PBS buffer. (TIF) [file pone.0088185.s003.tif]

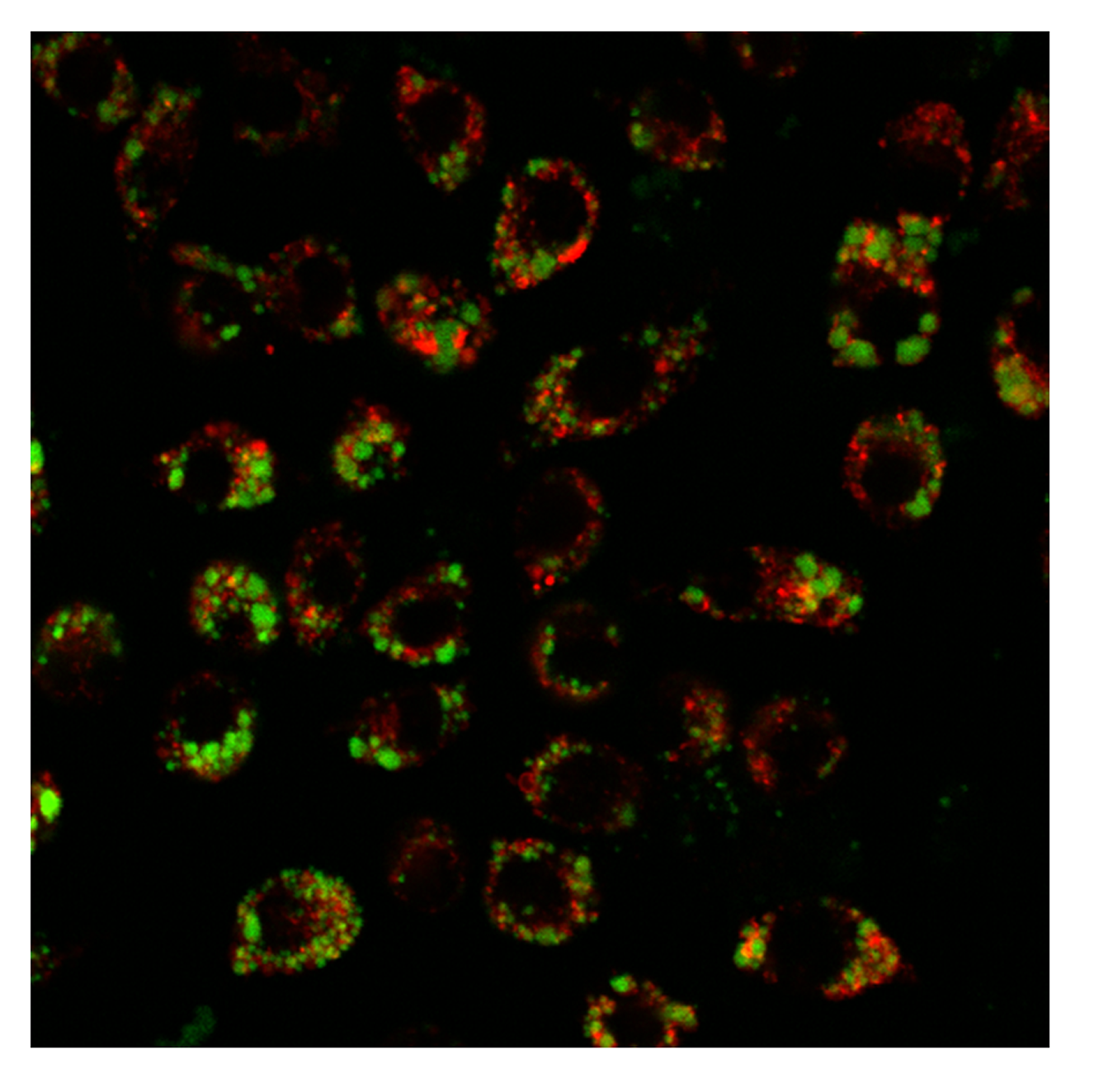

Supplement: Figure S4 — Magnified Figure 6f . (TIF) [file pone.0088185.s004.tif]

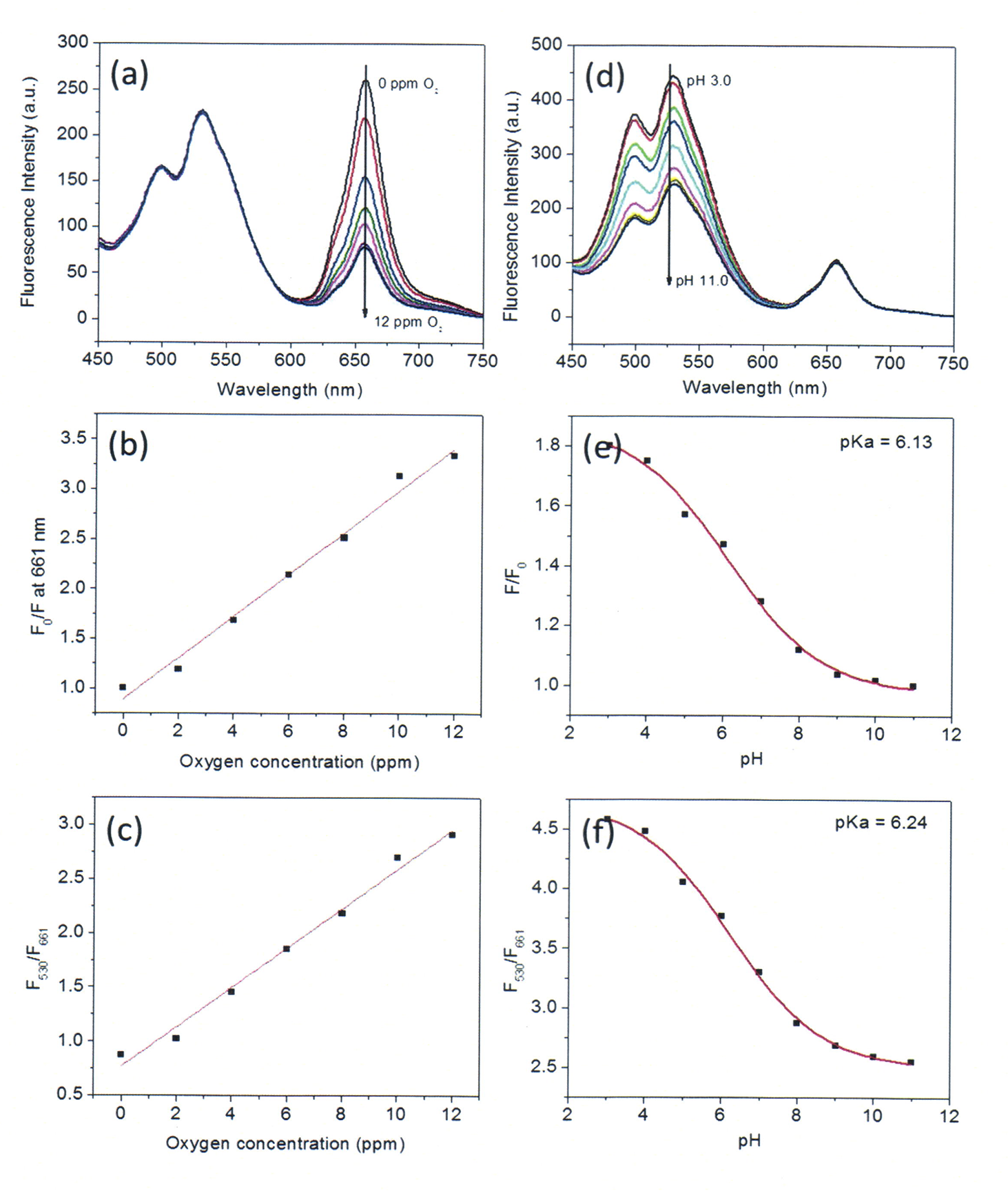

Supplement: Figure S5 — Response to dissolved oxygen in buffer. Fits of the Stern-Volmer plot with/without ratiometric calibration. (d–f) The emission profile of core-shell microgels changes as a function of pH. Intensity ratio variations with pH with/without ratiometric calibration. (TIF) [file pone.0088185.s005.tif]
