# Supplementary material for: Dually Fluorescent Core-Shell Microgels for Ratiometric Imaging in Live Antigen-Presenting Cells
Source: PLoS One. 2014 Feb 4;9(2):e88185. doi: 10.1371/journal.pone.0088185 (PMC3913776; doi:10.1371/journal.pone.0088185)
Supplement: Table S1 — Size of Poly(St- co -NIPAm) core (OS1) particles obtained by DLS at 25°C. In the systematic preparation of Poly(St- co -NIPAm) core particles, the dosages of St, NIPAm, and ABVA were kept constant as 1.0 g, 0.2 g and 0.01 mM, respectively. (DOC) [file pone.0088185.s006.doc]

| Tween 20 (g) | 0 | 0.01 | 0.05 | 0.10 | 0.20 |
| --- | --- | --- | --- | --- | --- |
| D_h_ (nm) | 474 | 356 | 259 | 172 | 94 |
