# Supplementary material for: Dually Fluorescent Core-Shell Microgels for Ratiometric Imaging in Live Antigen-Presenting Cells
Source: PLoS One. 2014 Feb 4;9(2):e88185. doi: 10.1371/journal.pone.0088185 (PMC3913776; doi:10.1371/journal.pone.0088185)
Supplement: Table S2 — Hydrodynamic mean diameters of the core-shell microgels (MS1) prepared with different NIPAm dosages. For all samples, the w/w percentage of BIS is 3%. (DOC) [file pone.0088185.s007.doc]

| NIPAm (g) | 0.2 | 0.4 | 0.8 |
| --- | --- | --- | --- |
| D_h_ (nm)-25^o^C | 448 | 628 | 886 |
| D_h_ (nm)-37^o^C | 194 | 265 | 305 |
