# Supplementary material for: Dually Fluorescent Core-Shell Microgels for Ratiometric Imaging in Live Antigen-Presenting Cells
Source: PLoS One. 2014 Feb 4;9(2):e88185. doi: 10.1371/journal.pone.0088185 (PMC3913776; doi:10.1371/journal.pone.0088185)
Supplement: Table S3 — Hydrodynamic diameters of the core-shell microgels (MS1) prepared with different BIS dosages. For all samples, the dosage of NIPAm is 400 mg. (DOC) [file pone.0088185.s008.doc]

| BIS (%) | 1% | 3% | 5% |
| --- | --- | --- | --- |
| D_h_ (nm)-25^o^C | 653 | 628 | 602 |
| D_h_ (nm)-37^o^C | 260 | 265 | 281 |
